# Inflammation in epileptogenesis after traumatic brain injury

**DOI:** 10.1186/s12974-016-0786-1

**Published:** 2017-01-13

**Authors:** Kyria M. Webster, Mujun Sun, Peter Crack, Terence J. O’Brien, Sandy R. Shultz, Bridgette D. Semple

**Affiliations:** 1 Department of Medicine (The Royal Melbourne Hospital), The University of Melbourne, Kenneth Myer Building, Melbourne Brain Centre, Royal Parade, Parkville, VIC 3050 Australia; 2 Department of Pharmacology and Therapeutics, The University of Melbourne, Parkville, VIC 3050 Australia

**Keywords:** Inflammation, Traumatic brain injury, Epilepsy, Post-traumatic epilepsy, Seizures, Cytokine, Interleukin, Astrocytes

## Abstract

**Background:**

Epilepsy is a common and debilitating consequence of traumatic brain injury (TBI). Seizures contribute to progressive neurodegeneration and poor functional and psychosocial outcomes for TBI survivors, and epilepsy after TBI is often resistant to existing anti-epileptic drugs. The development of post-traumatic epilepsy (PTE) occurs in a complex neurobiological environment characterized by ongoing TBI-induced secondary injury processes. Neuroinflammation is an important secondary injury process, though how it contributes to epileptogenesis, and the development of chronic, spontaneous seizure activity, remains poorly understood. A mechanistic understanding of how inflammation contributes to the development of epilepsy (epileptogenesis) after TBI is important to facilitate the identification of novel therapeutic strategies to reduce or prevent seizures.

**Body:**

We reviewed previous clinical and pre-clinical data to evaluate the hypothesis that inflammation contributes to seizures and epilepsy after TBI. Increasing evidence indicates that neuroinflammation is a common consequence of epileptic seizure activity, and also contributes to epileptogenesis as well as seizure initiation (ictogenesis) and perpetuation. Three key signaling factors implicated in both seizure activity and TBI-induced secondary pathogenesis are highlighted in this review: high-mobility group box protein-1 interacting with toll-like receptors, interleukin-1β interacting with its receptors, and transforming growth factor-β signaling from extravascular albumin. Lastly, we consider age-dependent differences in seizure susceptibility and neuroinflammation as mechanisms which may contribute to a heightened vulnerability to epileptogenesis in young brain-injured patients.

**Conclusion:**

Several inflammatory mediators exhibit epileptogenic and ictogenic properties, acting on glia and neurons both directly and indirectly influence neuronal excitability. Further research is required to establish causality between inflammatory signaling cascades and the development of epilepsy post-TBI, and to evaluate the therapeutic potential of pharmaceuticals targeting inflammatory pathways to prevent or mitigate the development of PTE.

## Background

Epilepsy is a common and debilitating consequence of traumatic brain injuries (TBI), with recurrent spontaneous seizures contributing to progressive neurodegeneration and greatly interfering with quality of life as well as increasing the risk of injury and death. Epileptogenesis, the neurobiological process by which epilepsy develops, occurs as part of the ongoing secondary injury events triggered by a brain insult, including neuroinflammation. Previous evidence from clinical and pre-clinical studies has suggested that aspects of the inflammatory response may also promote seizure activity itself (ictogenesis).

The aim of this review was to evaluate the published evidence regarding the role of inflammation in the development of post-traumatic epilepsy (PTE), drawing upon data from both clinical studies and experimental models. In particular, we summarize the current understanding of mechanisms by which neuroinflammatory mediators can influence neuronal excitability, either directly or indirectly. We focused in particular on three key signaling pathways which are known to be involved in TBI-induced secondary pathogenesis, and more recently, have been implicated in seizure activity and the process of epileptogenesis. Lastly, potential mechanisms underlying age-specific vulnerability to hyperexcitability and epileptogenesis are discussed. This review also acts to highlight knowledge gaps in the field, identifying key areas for future research. Ultimately, a mechanistic understanding of how neuroinflammation contributes to the development of epilepsy after brain injury may identify novel therapeutic targets, to reduce or prevent PTE for survivors of brain injuries.

## Traumatic brain injury and epilepsy

TBI is a major global public health problem and a leading cause of mortality and morbidity [1, 2]. It is particularly prevalent in childhood and adolescence, as a result of falls, inflicted trauma, sports-related injuries, and motor vehicle accidents. An earlier review of 11 studies examining TBI incidence in Australia, North America, and Europe estimated a median of 691 injuries per 100,000 population under 20 years of age [2]. Of note, children under the age of 5 had the highest incidence of Emergency Department admissions for TBI [2].

TBI is any insult to the brain from an external mechanical force, including penetrative or blunt trauma [1, 3]. These can include focal injuries, such as lesions caused by contusions or hemorrhages, or diffuse injuries, such as with traumatic axonal injury [4]. TBI involves a primary insult, defined as the immediate structural damage caused by the external mechanical force. This is followed by a secondary injury, which includes a myriad of neuropathological processes including excitotoxicity, neuroinflammation, oxidative stress, and apoptosis [1, 3, 5]. These secondary processes commence within minutes after TBI, can persist for months to years, and are thought to contribute to the expansion of tissue damage [6, 7]. The manifestation and severity of secondary injury processes can differ depending on injury type, severity, and individual factors [8]. The biomechanics and biochemical components of the physiological response to TBI have been reviewed in detail elsewhere [7, 9].

**Fig. 1:**
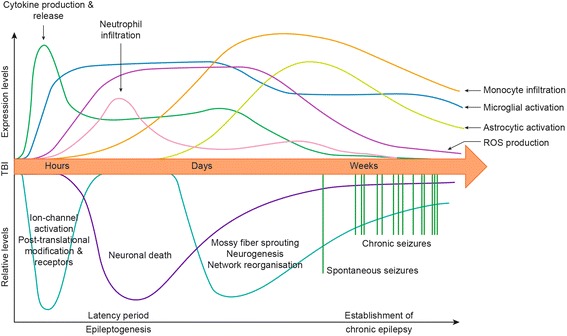
Summary of the progression of inflammatory factors and epileptogenesis after TBI. After TBI, epileptogenesis occurs after a latent period of months to years. Within hours after the injury, a myriad of cytokines are released at high levels which can continue for days. This is concurrent with activation of ion-channels and post-translational modifications of various receptors associated with neuronal excitation and inhibition, which can occur as early as minutes after the injury. Local immune cells are activated, and peripheral immune cells are also recruited to the area within hours to days. Neuroinflammation can persist for weeks after the injury, coincidental with widespread neuronal loss. In the later phase of epileptogenesis, processes such as neurogenesis and mossy fiber sprouting in the hippocampus contribute to an increasingly excitable neuronal environment. It may be weeks, months, or years before spontaneous seizures and the establishment of chronic and persistent epilepsy manifests

PTE is a common consequence of TBI, defined as spontaneous, recurrent, and chronic seizures following a head injury [10, 11]. Clinical diagnosis is often based upon one or more unprovoked seizures occurring later than 1 week after a TBI, as an indicator that epileptogenesis is occurring [12]. Epileptogenesis is the process by which epilepsy develops; that is, when an otherwise normally functioning brain becomes biased towards abnormal recurrent electrical activity, increasing the propensity to develop spontaneous recurrent seizures [13, 14]. It is thought to develop through three phases: (1) the initial trigger; (2) the latency period, during which the changes initiated in phase one cause a transformational bias in the brain towards epileptic activity; and (3) the onset of spontaneous seizures and the establishment of chronic epilepsy (Fig. 1) [15–18]. After TBI, the latency period may be many years in duration, and epileptogenesis is associated with ongoing secondary injury processes which can bias towards hyperexcitability [14].

The reported incidence of developing epilepsy after TBI ranges from 4.4 to 53%, depending on the population studied [12]. There are several factors that have been associated with a greater risk of developing PTE, including higher injury severity and a lower age-at-insult [10, 19, 20]. It has been estimated that 10–20% of children with severe TBI develop PTE, although the risk after injury has been reported at up to 60%, with the wide range of estimates most likely due to the large variation in severity, heterogeneity of the initial insult, and difficulties in diagnosis and follow-up [10, 20]. Children under the age of 5 may be at highest risk for early post-traumatic seizure development [10, 12, 21], with one study finding that this age group were more likely to have a seizure within the first week after injury (17%), compared to patients over 5 years of age with similar injuries (2%) [22]. In adults, the presence of acute intracerebral hematoma has been consistently associated with a higher risk of developing PTE, as are penetrative insults and depressed skull fractures [12, 22, 23].

Several neuropathological hallmarks have been associated with the development of PTE. An early and persistent increase in hippocampal excitability has been observed in both patients and animal models [24]. This net increase in excitability is thought to result from the selective loss of vulnerable inhibitory interneurons concurrent with the reorganization of excitatory circuitry [25, 26]. Recurrent excitatory circuitry in the hippocampal dentate gyrus may manifest as mossy fiber sprouting, where the axons of dentate granule cells form abnormal connections with neighboring neurons in response to a loss of CA3 pyramidal cell targets and hilar interneurons [27, 28].

The onset of PTE is also commonly associated with hippocampal sclerosis, involving the loss of pyramidal neurons, and concurrent reactive gliosis, consistent with temporal lobe epilepsy (TLE) [29]. An estimated 35–62% of patients with PTE have seizures originating from the temporal lobe [30, 31]. However, overlaying cortical regions have also been implicated in post-TBI epileptogenesis, as these regions may also exhibit neuronal loss, chronic neuroinflammation, and network reorganization resulting in spontaneous epileptiform activity [26, 32].

Clinical management and treatment of PTE is challenging, as seizures are commonly resistant to existing anti-seizure drugs (ASDs) [10, 20, 33, 34]. Classical ASDs, such as phenytoin, carbamazepine, valproate benzodiazepines, are ineffective in reducing or preventing PTE [34–36]. While early post-injury prophylaxis with ASDs may reduce or prevent early post-injury seizures [37], there is little evidence to indicate that these treatments can be disease-modifying and prevent the development of PTE or spontaneous unprovoked seizures long-term [38]. Once a patient has developed seizures, polypharmacy is often employed in an attempt to control the seizures, yet a significant proportion of TBI patients who develop epilepsy will develop drug-resistance, defined as a failure to achieve seizure cessation after trialling more than two tolerated and appropriate ASD treatments [33, 34]. However, the use of multiple ASDs simultaneously could have unpredictable consequences due to potential interactions with various secondary processes and the reduced cerebral perfusion commonly present after TBI [20]. Seizures are particularly detrimental during periods of brain development as they can cause permanent adverse effects, including cognitive deficits [39, 40]. Due to increased susceptibility to post-traumatic seizures after an early age-of-insult, as well as inherent difficulties in controlling or treating PTE, further research is needed to understand the mechanisms that contribute to the generation of post-traumatic seizures, particularly in pediatric age groups.

## Neuroinflammation after TBI

Inflammation is a central component of the secondary injury after TBI, and the subject of intense research as a promising target for treatment. In healthy tissue, inflammation typically acts to combat invading pathogens and preserve the health of the tissue [41]. However, in pathological conditions such as trauma, inflammation can also function as a reactionary system to either aggravate or ameliorate tissue damage [42, 43]. Increased neuroinflammation after TBI has been associated with poor outcomes and progression to various sequelae including neurodegenerative diseases [43–47].

The main hallmarks of the cerebral inflammatory response after TBI include blood-brain barrier (BBB) dysfunction, edema, microglial, and astrocytic activation and migration, the release of inflammatory factors such as cytokines and the recruitment of blood-derived leukocytes into brain parenchyma [48]. Neutrophils, recruited from the peripheral circulation within hours after TBI, mediate early pathogenesis by promoting edema and oxidative stress, and the production of inflammatory cytokines and neurotoxic proteases [49, 50]. Cytokines can be released rapidly after injury as they are synthesized and stored locally by neurons and glia [48].

Under physiological conditions, the BBB is a highly stringent barrier between vessels and brain tissue, which mediates the transport of blood components such as immune cells into the brain [51]. After TBI, this barrier can be compromised, allowing peripheral inflammatory cells into the brain and the injured area [52]. Chemoattractant cytokines, called chemokines, further facilitate the recruitment and transmigration of inflammatory leukocytes [53]. By their actions at the BBB as well as direct chemoattraction, chemokines including CXCL8 and CCL2 (also known as monocyte chemoattractant protein-1) are key mediators in the migration of neutrophils and monocytes, respectively, to the site of injury [54].

Once in the brain, these cells release a plethora of inflammatory cytokines, chemokines, and reactive oxygen species (ROS) to perpetuate inflammation and oxidative stress in the injured brain. Both clinical and experimental studies have demonstrated a pronounced elevation of many cytokines after TBI, including tumor necrosis factor (TNF-α), transforming growth factor-β (TGF-β), and interleukin-1β (IL-1β), -6, and -10, with downstream activation of intracellular signaling cascades involving nuclear factor Kappa-light-chain-enhancer of activated B cells (NF-κB) [1, 44, 48, 55–57]. Released cytokines in turn can recruit additional blood-borne neutrophils and monocytes into the injured tissue, propagating the inflammatory cascade.

Inflammation in the brain has a duality in function after injury, which manifests through the actions of many different cell types. For example, microglial activation is integral in tissue repair, surveillance of pathogenic factors and host defense [58]. However, activated microglia can also release cytotoxic factors such as ROS to induce oxidative stress [59–61]. Astrocytes can promote tissue repair in the central nervous system (CNS) through the release of insulin-like growth factor [47], but are also implicated in the perpetuation of inflammation by an over-production of cytokines such as IL-6 [62, 63], as well as modulation of the BBB and neuronal function to promote excitability and seizure production [64].

Cytokines themselves also have a complex role after TBI, as experimental studies have yielded conflicting findings of both deleterious contributions and participation in repair processes after CNS insult [48]. One cytokine that displays such paradox is TNF-α, which has been associated with increased neurological damage, including demyelination and BBB breakdown, in several experimental models of TBI [65, 66]. However, increased levels of TNF-α may conversely have a neuroprotective function in the late stages of inflammation post-TBI, at 2–4 weeks after the injury, as suggested from a mouse model of TBI [56]. The varied roles of inflammatory mediators in the pathological environment after TBI is likely dependent on many different factors, including timing of release, the location and cell types involved, the differing physics of protein-protein interactions of cytokines, and their relative amounts. The multifarious dynamics of this response may contribute to the progression of a chronic state of damage, leading to the myriad of secondary consequences of TBI, including PTE.

## Inflammation in epileptogenesis

The long-standing concept that seizures result from an imbalance between reduced γ-aminobutyric acid (GABA)-ergic inhibition and enhanced glutamatergic excitation [67, 68], based upon the presence of large amplitude EEG discharges during the seizure event itself, is an over-simplified of a very complex network response. While excessive glutamatergic excitation has historically been considered of as the precipitating factor for a focal seizure, there is a lack of strong data to support this hypothesis. Instead, paradoxically, accumulating evidence indicates that increased synchronised GABAergic interneuronal activity is sufficient to disrupt neuronal networks and initiate the transition from interictal to ictal activity resulting in focal seizures [69]. The recruitment of neighboring neurons and subsequent seizure progression is then hypothesized to be mediated by an elevation in extracellular potassium [70]. Adding to the complexity of network-based activity, both excitatory and inhibitory roles of GABA and glutamatergic neurons have been reported, and a range of extrasynaptic as well as synaptic neurotransmitter receptors and ion channels have been implicated in seizures, in addition to those traditionally implicated, such as NMDA and GABA_A_ receptors [71].

However, strong evidence also implicates a role for inflammation in seizure pathologies [45]. Seizure activity readily induces an inflammatory response, including the activation of microglia and production of pro-inflammatory cytokines [47, 63]. More importantly, experimental data has suggested that inflammatory mediators may initiate or trigger early seizures, preceding the onset of diagnosed epilepsy. For example, systemic inflammation by injection of bacterial lipopolysaccharide results in a lowered seizure threshold [72]. In the next sections, we will review clinical and experimental evidence suggesting an inherent link between inflammatory signaling, neuropathology, and seizure activity in the injured brain, as a likely mechanism of importance in the development and progression of PTE.

### Seizures increase inflammation

Experimentally, induction of a seizure induces the rapid activation of glial cells in surrounding parenchyma, which respond by the production and release of inflammatory molecules [73]. Much of the research that has shown an increase in inflammation after seizures have used experimental models of status epilepticus. This involves the administration of a chemical or electrical pro-convulsant stimulus to create a sustained seizure event (the initial insult) followed by a latency period before the onset of spontaneous recurrent seizures to model epilepsy [74, 75]. In these experimental models, the inflammatory response displays a distinct temporal profile after induction, characterized by the early activation of astrocytes and microglia followed by BBB breakdown and neuronal activation [63, 76, 77]. In addition to the investigation of protein release, microarray analysis of gene transcripts have also demonstrated an upregulation of inflammatory genes [78]. Specific cell surface toll-like receptors (TLR’s), which respond to a range of inflammatory cytokines and other stress-related factors, are highly upregulated after pilocarpine-induced seizures on forebrain microglia of adult mice [77]. Simultaneously, a robust increase in cytokine levels has been observed in both chemically and electrically induced experimental models of epilepsy in adult rodents [77, 79, 80]. For example, IL-1β is expressed at low levels in a healthy brain, but is robustly upregulated for up to 60 days after the induction of self-sustaining limbic status epilepticus in rodents, a model using hippocampal electrical stimulation [76]. TNF-α and IL-6 are also rapidly upregulated after status epilepticus, peaking within 30 min of seizure onset and remaining elevated for up to 72 h in rats that progressed to spontaneous seizures [76].

These experimental findings that seizures result in inflammation are confirmed by evidence in the clinical setting. Analysis of cerebrospinal fluid (CSF) from newly diagnosed adult patients with tonic-clonic seizures detected an upregulation of IL-6 and IL-1 receptors (IL-1Rs) [81, 82]. Matched serum samples revealed a higher levels of IL-6 compared to in the CSF, suggesting that these cytokines likely originated in the brain [82]. High levels of cytokines including IL-1β and high-mobility group box protein-1 (HMGB1) have also been identified in neurons and glia of surgically resected epileptic tissue [83]. Together, these findings indicate that neuroinflammation is a common consequence of seizure activity.

### Inflammation contributing to seizures

Accumulating evidence suggests that neuroinflammation is also a contributor to epileptogenic pathology after TBI [45, 63, 84]. In particular, experimental models have demonstrated that glial cell activation and recruitment and the synthesis of inflammatory factors, may precede and/or occur concurrently with epileptogenic events [85, 86]. For example, in a rodent model of experimental TBI, a reduced threshold to electroconvulsive shock-induced seizures was reversed when minocycline, a tetracycline antibiotic known to inhibit brain infiltration of monocytes and microglia, was applied [87, 88], implicating both microglial activity and pro-inflammatory cytokines in post-traumatic seizure activity.

Much of the evidence for a role of inflammation in epileptogenesis has focused on the effect of cytokines in seizure susceptibility. Cytokines can act as classical neurotransmitters through receptor modulation and phosphorylation at the neuronal membrane [89]. Models of chronic inflammation, such as transgenic mice systemically overexpressing IL-6 or TNF-α, can reduce seizure threshold and predispose the brain to seizure induced-neuronal loss [90, 91]. Indeed, inflammatory signaling may promote the loss of GABAergic neurons in the hippocampus, resulting in an increased propensity for seizures due to a reduction in synaptic inhibition [92].

N-methyl-D-aspartate (NMDA) receptors play a critical role in the glutamatergic system to contribute to neuronal excitability, and previous evidence suggests both direct and indirect interactions between these receptors and cyokines [93]. Cytokines have been found to inhibit the uptake of glutamate by astrocytes in culture [94] and modulate excitatory neurotransmission in the brain through NMDA and alpha-amino-3-hydroxyl-5-methyl-4-isoxazole-propoinate (AMPA) receptors [95, 96]. For example, IL-1β produced by microglia can enhance NMDA-mediated Ca^2+^ currents through cell surface type 1 IL-1R (IL-1R1) co-localized on pyramidal cell dendrites [89]. Pre-synaptic NMDA receptors are agonists for Ca^2+^-mediated glutamate release, and when activated by inflammatory factors such as IL-1β and HMGB1 can cause an excess of intracellular Ca^2+^ leading to an extracellular hyperexcitability and excitotoxicity [95]. Several other cytokines including TNF-α and IL-10 have also been associated with the regulation of seizure duration in experimental kindling models [97, 98]. Though these correlations have been seen in multiple studies with different models, the mechanisms underlying the relationship between the inflammatory environment and epileptogenesis, particularly in the context of brain injury, remain still poorly understood.

There is limited clinical data to confirm a cause-and-effect link between inflammation and the pathophysiology of epilepsy, however increasing evidence supports this hypothesis. Many studies have now demonstrated that early exposure of the brain to immune responses can have varied and persistent consequences on adult physiology [99–102]. Febrile seizures (FS) and febrile status epilepticus in children is a risk factor for developing epilepsy later in life [103], which may be induced by fever often associated with inflammation and infection [47]. Although the mechanisms underlying FS remains unclear, it is thought that cytokines play a key role in its development [104]. One study has reported specific polymorphisms in the promoter region of cytokine genes, including IL-1β, in children with FS compared to controls [105]. Such genetic variation may influence the production of IL-1β in both healthy tissue and after injury or stimulus [106], and similar polymorphisms have also been observed at a high frequency in patients with TLE [107].

**Fig. 2:**
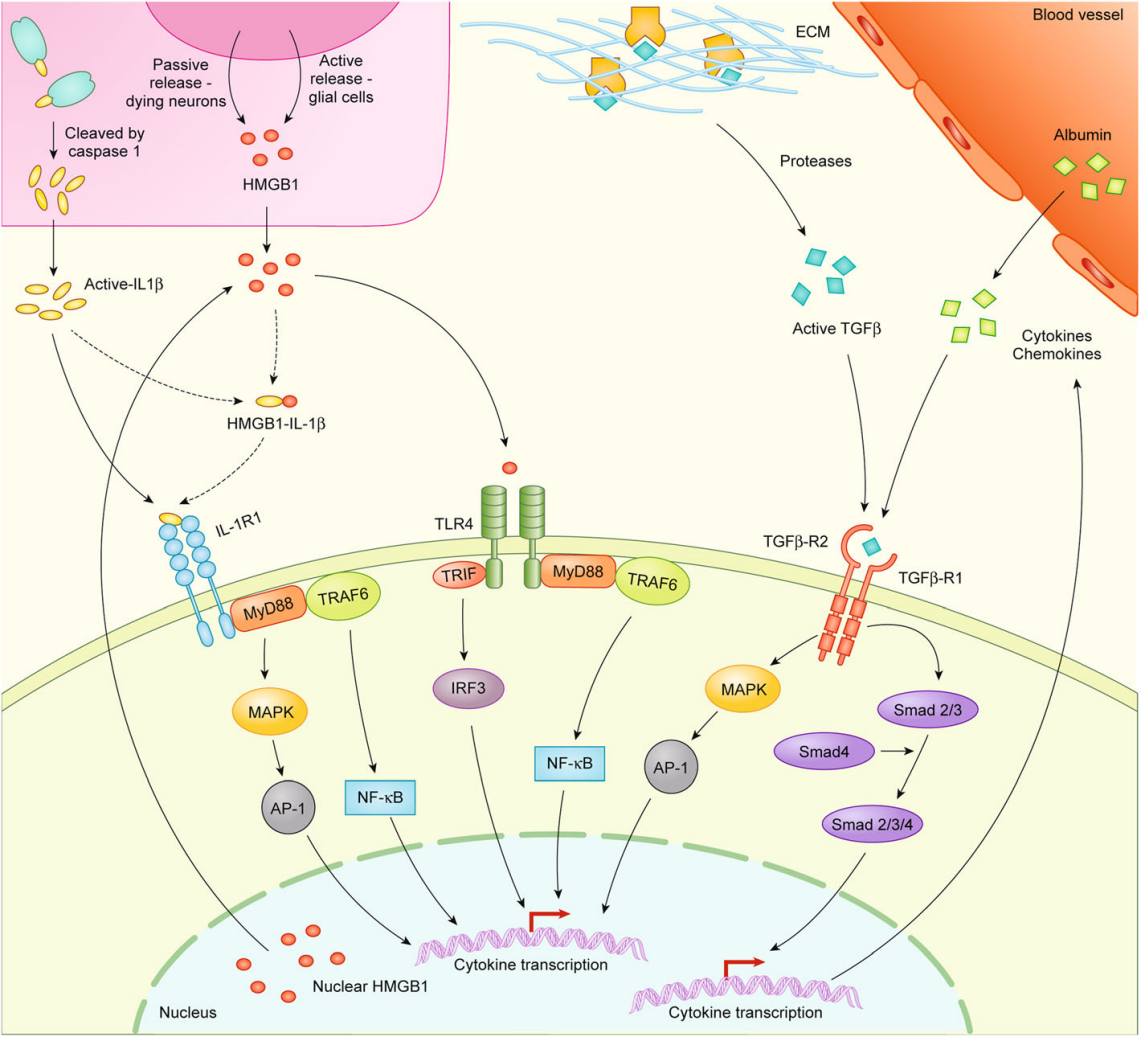
Summary of three key signaling cascades that may mediate the link between inflammation and epileptogenesis. HMGB1, IL-1β, TGF-β, and serum albumin have varied release mechanisms from multiple cell types in order to activate their signaling pathways. After injury, HMGB1 may be passively released from necrotic neurons to the extracellular space, or released actively from activated microglia and astrocytes. HMGB1 can bind to multiple receptors on many different cell types, such as TLR4, which can activate MyD88 independent pathways such as the phosphorylation of interferon regulatory transcription factor 3 (IRF3) leading to the transcription and release of interferons-α and -β, as well as other interferon-induced genes. HMGB1-TLR4 can also activate NF-κB signaling both directly or via TNF receptor-associated factor 6 (TRAF6). This can lead to a rapid nuclear transcription of various immune-related processes, as reviewed elsewhere [250]. Caspase-1 mediates cleavage of inactive pro-IL-1β to active IL-1β, allowing for its relocation into the extracellular space, where IL-1β can bind to IL-1R1 either directly or in complex with HMGB1. The IL-1β/IL-1R1 complex can then induce NF-κB signaling via TRAF6 or activate MyD88-dependent MAPK signaling, which has been linked to the production of various neurotoxic molecules. TGF-β is released in an inactive form from cells and binds to the extracellular matrix. Proteases, released after injury, cleave the inactive protein to active TGF-β, which is able to bind to the two TGF-β receptors. Mechanical breakdown of the BBB allows serum albumin into the extracellular space, where it can also bind to TGF-β receptor1 and receptor2, which signal via Smad complex proteins or MAPK signaling pathways, respectively, to regulate the immune response. This pathway has also been implicated in post-translational changes to a variety of voltage-dependent ion channels implicated in changes to neuronal excitability [94]

Recently, the hypothesis of glial functions playing a pivotal role in biasing the neuronal network towards an epileptogenic environment has been gaining traction [108, 109]. In particular, interactions between neurons, glia, and the inflammatory mediators IL-1β, HMGB-1, and TGF-β, have been implicated in promoting seizure susceptibility, as described below. The main signaling pathways implicated in the proposed link between inflammation and epileptogenesis of these three mediators is summarized in Fig. 2.

## IL-1β/IL-1R signaling in TBI and PTE

IL-1 is a family of pro-inflammatory cytokines that act as key mediators of the innate immune response [110]. The IL-1 family consists of 7 agonists (e.g., IL-1α and β) and 3 receptor antagonists [110], and amongst these the IL-1β isoform has been the most commonly studied in the brain injury and epilepsy settings. In the CNS, IL-1β can be produced by a range of cells including microglia, astrocytes, endothelial cells (EC), neurons, and peripheral leukocytes upon infiltrating into the brain [111–113]. IL-1β exerts its action on multiple cell types primarily via IL-1R1 [114–119]. This initiates intracellular signaling via NF-κB transcription factor, p38 mitogen-activated protein kinase (MAPK), or other factors [118, 120]. Several studies have demonstrated that IL-1β binding to IL-1R stimulates immune cell activation and induces the production of neurotoxic molecules [114, 115, 117–119].

There are several lines of evidence implicating IL-1β in the development of PTE. Firstly, IL-1β is rapidly and highly upregulated following experimental and clinical TBI. In rodent models, Il-1β expression is upregulated as early as 1 h post-TBI [121, 122] and peaks between 12 and 24 h [54, 123, 124]. This response may then persist for several months post-injury [125]. Consistent with experimental findings, analysis of protein, and gene expression in post-mortem brain tissue from TBI patients found that IL-1β was upregulated in individuals who died 6–122 h post-injury [126]. This is consistent with reports of elevated IL-1β in the CSF and serum of severe TBI patients [127, 128], correlated with poor outcomes in both children and adults [19, 129]. Thus IL-1β levels are elevated during the period of secondary injury after TBI, which has been postulated to also be an important time period for the epileptogenic process [130]. Notably, numerous studies indicate that modulating IL-1β signaling is broadly beneficial in experimental TBI models. Treatment with an IL-1β neutralizing antibody alleviated TBI-induced microglial activation, neutrophil infiltration, cerebral edema, and cognitive deficits in mouse models of TBI [131, 132]. In line with this finding, IL-1R1 deficient mice also showed decreased cerebral edema and leukocyte infiltration following TBI [133], and post-injury administration of an IL-1R1 antagonist attenuated neuronal cell death and cognitive dysfunction in rats [134]. A phase II clinical trial employing an IL-1R antagonist treatment (100 mg/day subcutaneously administered for 5 days) was recently conducted in patients with severe diffuse TBI, demonstrating safety penetration into the plasma and brain extracellular fluid, and an alteration of the immune profile [135].

One of the key clinical studies linking IL-1β with PTE was conducted by Diamond and colleagues, who examined whether genetic variation in the IL-1β gene and CSF/serum IL-1β ratios correlated with PTE development in a cohort of 256 patients with moderate to severe TBI [136]. Serum and CSF were collected from a portion of subjects within the first week post-injury, and IL-1β levels were assessed in relation to the later incidence of PTE. Further, IL-1β tagging and functional single nucleotide polymorphisms (SNPs) were genotyped to evaluate its association to PTE. Elevated CSF/serum IL-1β ratios were found to be associated with increased risk of PTE, and one of IL-1β SNPs, rs1143634, showed an association between the heterozygote genotype and increased PTE risk [136]. Further research is required to determine the contribution of genetic variability to IL-1β function and how this may influence the inflammatory response after TBI.

IL-1β has also been linked to other epilepsies in addition to PTE. For example, IL-1β may play a role in epileptogenesis that follows FS. Specifically, IL-1β levels were found to be acutely upregulated in rats after prolonged FS, and IL-1β levels remained elevated only in rats that developed spontaneous limbic seizures after prolonged FS [137]. In another study, IL-1R1 deficient mice were found to be resistant to experimental FS [138].

There are a number of possible mechanisms by which IL-1β may contribute to PTE. IL-1β can modulate neuronal hyperexcitability through Ca^2+^, glutamatergic, and GABAergic pathways [139]. By acting on glia cells, IL-1β mediates astrocytes and microglia activation, formation of a glial scar, and the release of neurotoxic mediators to promote cell loss, features which are associated with epileptic foci in the brain [140]. Furthermore, IL-1β signaling may promote epileptogenesis by enhancing BBB permeability and enhancing the recruitment of peripheral leukocytes into the brain [141–144]. There are many proposed mechanisms of IL-1β signaling in areas that are still poorly understood, and future research into these may reveal the way in which they interact to increase the risk of epileptogenesis.

Although less commonly studied, other members of the IL-1 family have also been investigated in the context of TBI and epilepsy. For example, an upregulation of IL-1α has been reported in brain tissue following experimental TBI [145, 146]. In the clinical setting, peripheral blood mononuclear cells collected from epilepsy patients exhibited greater production of IL-1α in response to stimulation in vitro compared to cells from non-epileptic controls [147]. However, no association has been found between gene polymorphisms of IL-1α and TBI outcomes [148], nor IL-1α and seizure pathogenesis [149]. Taken together, there is much accumulating experimental and clinical evidence implicating IL-1 cytokines in epileptogenesis, however further studies are needed to delineate their precise roles and whether therapeutically targeting them can mitigate PTE.

## HMGB1/TLR4 signaling in PTE

HMGB1 was originally identified as a ubiquitous, highly evolutionarily conserved, chromatin-binding protein, most often found in the cell nucleus of healthy tissues [150–152]. Discovered in 1973, and named due to its ability to migrate quickly during gel electrophoresis [153], HMGB1 participates in the formation of nucleosomes and is important in the regulation of gene transcription [151, 154, 155]. More recently, HMGB1 has been identified as a damage-associated molecular pattern (DAMP) [150], which are molecules that are able to initiate or perpetuate inflammation. Complete gene deficiency of HMGB1 is lethal during early postnatal life, indicating its essential role in transcriptional control [156]. A 98% homology between human HMGB1 and mouse HMGB1 enables clinically relevant experimental investigation through animal models [157].

HMGB1 has two modes of cellular release in injured tissue. Immediately after injury, necrosis allows a passive release of significant amounts of HMGB1 from the nucleus into the extracellular space [158]. HMGB1 may undergo post-translational oxidation and acetylation, resulting in its active release by immune cells in response to various cytokines including itself, allowing a powerful positive feedback loop during inflammation [157, 159–161]. In addition, HMGB1 may be released upon activation by a wide range of cells in the CNS including neurons, microglia, macrophages, monocytes, natural killer cells, dendritic cells, ECs, and platelets [162].

High levels of HMGB1 have been found in epileptogenic tissue resected during surgery [83, 163], implicating a role for this DAMP in neuronal hyperexcitability. Experimental evidence from an animal model of temporal lobe epilepsy suggests that HMGB1 contributes to seizure activity and epilepsy [164]. One previous study by Maroso and colleagues demonstrated that intracerebral injection of HMGB1 in wild type mice increased seizure activity in response to a stimulus [165]. This pro-convulsant effect of HMGB1 is most likely mediated through one of its key signaling receptors, TLR4, as a comparable increase in seizure susceptibility was not seen in non-functional TLR4 mutant mice [165]. TLR4 mutant mice were also found to be intrinsically resistant to seizures as compared to wild type mice [165]. Interfering with the binding of HMGB1 to TLR4 has been shown to reduce both seizure frequency and onset. For example, TLR4 antagonists and BoxA, a competitive antagonist to HMGB1 comprising of the BoxA domain of the protein, have been reported to reduce and even inhibit epileptic activity that is resistant to standard ASDs in an animal model of temporal lobe epilepsy [166].

In another recent study, the mechanism by which HMGB1 signaling promotes neuronal hyperexcitability and seizure activity was found to be through an increase in NMDA receptor function in TLR4-expressing hippocampal neurons [167]. This effect was dependent on the oxidation of HMGB1 characteristic of its active release from immune cells, and was associated with high levels of NMDA-induced excitotoxic cell death [167]. Because the inflammatory environment after TBI involves the rapid production of ROS and free radicals [168, 169], there is a physiological preference towards the extracellular oxidation of HMGB1, thus increasing TLR4-mediated augmentation of NMDA functionality and promoting seizure susceptibility [167].

Downstream of HMGB1, TLR4 mRNA expression is upregulated in response to brain insult [170], consistent with upregulation of this receptor during seizure activity in experimental models of epilepsy [165]. TLR4 is able to transmit signals via both myeloid differentiation primary response gene 88 (MyD88) dependent and independent pathways [171]. It is through the MyD88 dependent pathway that TLR4 is able to activate NF-κB, which may be responsible for increasing pro-inflammatory cytokine expression to augment the inflammatory response [172, 173]. This pathway has generated great interest recently, and with the potential to modulate epileptic activity, even in drug-resistant epilepsy, further research is needed to confirm the potential of this pathway as a target for therapeutic intervention in the development of PTE.

Previous research has also suggested that HMGB1 is able to enhance inflammation through forming a complex with different cytokines, including IL-1β [174]. HMGB1 bound to IL-1β has been isolated from cell cultures when co-incubated [174]. Studies of joint inflammation in animals have also shown that when HMGB1 is present with IL-1β there is an enhanced inflammatory response, most likely through action on with IL-1 receptor 1[175], Whilst this complex has yet to be identified in brain tissue, further research is needed into this pathway as it may play a vital role in extending a bias towards epileptogenesis.

By contributing to acute and chronic seizures, both directly and indirectly, the HMGB1-TLR4 axis is therefore a promising target for TLEs that are resistant to ASDs [165, 167]. This is important in the context of TBI, as seizure pathology can begin as early as the day of a brain injury [176]. Together, these data support a key role for HMGB1 in neuronal hyperexcitability and implicate the HMGB1-TLR4 signaling pathway as a potential therapeutic target to modulate post-traumatic epileptogenesis.

## TGF-β/albumin involvement in PTE

Vascular dysfunction has been associated with many types of focal and acquired epilepsies, including PTE [177]. BBB dysfunction is also a common feature of TBI in both patients and experimental models [6, 178]. Vascular dysfunction after TBI, in particular the localized breakdown of the BBB surrounding focal regions of tissue damage, is hypothesized to trigger a series of epileptogenic processes [179]. For example, increased permeability of the BBB is evident by magnetic resonance imaging with gadolinium and co-localized with the focal epileptogenic region in patients with PTE [180, 181].

A proposed mechanism of BBB breakdown in this context is through the TGF-β/albumin-mediated signaling pathway. In the laboratory, experimental opening of the BBB in the rodent neocortex was found to trigger epileptogenesis, which was recapitulated by exposure of the brain to serum albumin [182]. Albumin has been shown to induce the production of excitatory synapses experimentally both in vitro and in vivo [183]. Serum albumin in the rodent brain has been shown to result in hypersynchronized responses to electrical stimulation [184], analogous to those observed in animal models of epilepsy [185, 186]. The formation of epileptiform activity after albumin exposure is delayed, suggesting that the mechanism of action is complex rather than a direct effect by albumin [184]. One possible mechanism which has been receiving scrutiny is the activation of astrocytes by serum albumin [109, 187], activating a TGF-β receptor-mediated signaling cascade [64, 182, 184].

TGF-β is a pleiotropic cytokine involved in various cellular processes, including cell growth, differentiation, morphogenesis, apoptosis, and immune responses through intercellular communication in many different cell types [188–190]. Signaling is mediated by binding of TGF-β to two serine threonine kinase receptors, which when activated cause the phosphorylation of the Smad protein complex and the p38 MAPK pathway [191]. A role for TGF-β has been implicated in many different CNS diseases, related to its upregulation in Alzheimer’s disease, multiple sclerosis, ischemic brain injury, and TBI [192]. TGF-β is thought to have neuroprotective properties, and is associated with both microglial activation and the wound healing response [193]. Paradoxically, TGF-β also appears to contribute to excitotoxicity, adding to the dual role of inflammation which is dependent on the context and cell types involved [194]. Further research into the variation in response from this pathway is needed, as this may reveal insights into the dual role of inflammation and how to bias the brain towards neuroprotection rather than neurodegeneration.

The binding of albumin to TGF-β receptor has been characterized experimentally in rodent models of BBB disruption, resulting in the activation of TGF-β signaling [184]. The induction of experimental BBB dysfunction to induce epileptiform activity can be prevented through blockage of albumin binding to TGF-β receptors [184]. Much of the published literature has focused on albumin’s interaction with the TGF-β signaling pathway in disease models, but there is some suggestion that TGF-β may also play a role in PTE from animal models with similar pathologies. In rodent models of epilepsy, TGF-β is reportedly upregulated in both neurons [195] and hippocampal astrocytes [196]. The action of TGF-β in astrocytes following exposure to albumin has also been shown to induce pro-ictogenic cytokine production, resulting in increased neuronal excitability in experimental models [197]. TGF-β has also been implicated experimentally in excitatory synaptogenesis in a post-injury epilepsy model [183]. As neuronal reorganization and synaptogenesis are hypothesized to be a potential mechanism underlying chronic seizures and are well-documented consequences of CNS trauma, this action of TGF-β may be critical for the progression of brain injury to epilepsy [183, 198, 199]. In summary, the actions of TGF-β in the injured and epileptic brain remain poorly understood, but the potentially paradoxical behavior of this cytokine warrants further investigation.

## Age-specific vulnerability to PTE

Neuroinflammation is a key aspect of secondary injury that can vary according to the stage of brain development, which may underlie differences in clinical outcomes between patients who suffer a TBI during childhood and patients who suffer a TBI during adulthood [200]. Many studies now suggest that the early postnatal brain has an enhanced propensity for inflammation, described as a ‘window of susceptibility’ [200–202]. Early support for this hypothesis came from experimental evidence that 3-week old (juvenile) rats showed a higher susceptibility to IL-1β-induced BBB breakdown compared to adult rats [203, 204]. These observations are most likely not due to an immaturity of the BBB integrity at a younger age, as the tight junctions that maintain the BBB are fully developed from prenatal stages [205], but rather to a unique global chemokine expression profile that is distinctly different to the adult CNS [200]. In experimental autoimmune encephalomyelitis, a model of multiple sclerosis that shares some of the inflammatory processes of PTE, many chemokines involved in the recruitment of T cells and monocytes into the brain are robustly upregulated to 2–6-fold higher in juveniles compared to adults, including CCL2, CCL3, and CCL6 [200, 206–208]. There is also some evidence in the clinical context that the response of the immune system in the brain after a pathogenic challenge also differs between adults and children, with children presenting with higher production of IL-1 and IL-10 from peripheral blood monocytes after pathogenic stimulation [209]. Several cytokines have been detected at elevated levels in both CSF and serum of children after TBI, including IL-1α, IL-6, IL-12, and TNF-α, though how this response may correspond to the clinical differences at different ages after TBI are still relatively underinvestigated [210–212].

Microglia present differently within the immature brain compared to adults. During early postnatal development, their morphology is distinct, with fewer processes than those in the adult brain [213], and in an experimental pathogenic model, neonatal brain injury induced a markedly reduced activation compared to the robust activation seen in adult brain tissue [214]. Activated microglia typically adopt a phagocytic morphology and are associated with neuronal death, which is thought to be a critical aspect of normal brain development [201, 215]. There are also more circulating macrophages in the developing brain compared to adults under basal conditions associated with dying neurons and glia, particularly in the corpus callosum [213, 216]. Neutrophil infiltration into brain parenchyma after injury differs across ages, with a much higher level of infiltration detected experimentally in postnatal day 7 (p7) rats than in adults [214]. This neutrophil infiltration can persist 2–3 days after a pathogenic challenge and was found to be concurrent with damage to vasculature [203, 204, 214].

Increased seizure susceptibility in the immature brain may result from several contributing factors. Glutamate is the primary neurotransmitter in both the adult and developing brain [217], yet there is low expression of the glutamate type 1 transporter, therefore, clearance from the synaptic cleft is markedly slower compared that in the adult brain [218]. GABA receptor action is normally inhibitory in the adult brain, but is predominantly excitatory during early brain development due to high intracellular concentrations of chloride as a result of differential developmental expression of specific chloride ion transporters [219–221]. During early cortical development, experimental models have shown that GABA is predominantly depolarizing, progressing through childhood to the hyperpolarizing state found in the healthy adult brain [222, 223]. A recent study of experimental mesial temporal lobe epilepsy found a depolarizing role of GABA receptor, which was highly upregulated in surviving epileptic neurons [224], suggesting that the early neurodevelopmental environment may be more vulnerable to hyperexcitability after an insult. NMDA receptors are also more permeable to Ca^2+^ during development and desensitize more slowly [225]. In addition to a shorter post-ictal refractory period compared to the adult brain [226, 227], this inherently increased excitability of the pediatric brain may underlie an increased propensity for PTE after TBI during early childhood.

However, other studies have noted that the immature brain may conversely show resistance to seizure-related pathology; for example, chemical stimulation to experimentally induce sustained hyperexcitability is more difficult in the immature brain [39]. Infant brains may also be more resistant to excitotoxic-induced cell death than the adult brain [228]. Due to the apparent paradox of age-related differences, further study pertaining to the specific excitability of the developing environment is needed, particularly in the context of brain injury.

### Inflammation in pediatric PTE

As a result of the abovementioned evidence of age-specific responses to both inflammatory stimuli and seizures, a potential link between neuroinflammation and seizure susceptibility particularly during early childhood is under intense scrutiny. Experimental studies have implicated a role for neuroinflammation in increased seizure susceptibility in the immature brain; for example, exposure to several chemoconvulsant challenges revealed a lower seizure threshold in immature rats compared to adults [102]. The authors attribute this response to increased TNF-α or IL-1β production by activated microglia in the hippocampus in immature rats, which was associated with cognitive deficits and widespread neuronal loss [102, 229]. Changes in seizure threshold caused by neuroinflammation in the pediatric brain are associated with long-term alterations in glutamate receptors in the hippocampus, and a resultant increase in neuronal network excitability [229, 230].

In addition to the younger brain having an increased propensity for an enhanced neuroinflammatory response, age-specific differences in important epileptogenic factors have been identified. One such difference is NMDA receptor density, which has been shown experimentally to peak at p28 at approximately 160% of adult levels [231]. NMDA receptors also have a different subunit composition in the developing brain compared to the adult brain, resulting in different functional properties [232, 233]. Due to these changes, NMDA receptors are able to depolarize more easily in the presence of glutamate, resulting in a longer duration of excitatory postsynaptic currents [218, 234].

## Therapeutic targeting of post-traumatic inflammation to prevent PTE

TBI induces a neuroinflammatory cascade in the brain, leading to persistent and perpetuating neurodegeneration and likely contributing to an increased risk of initiating epileptogenesis, resulting in the development of PTE. In the context of the ongoing pursuit of novel therapeutic targets to prevent PTE, both clinical and experimental studies provide compelling evidence implicating IL-1β and HMGB1 as pivotal players in the cascade. Due to their early and varied role in the neuroinflammatory cascade after injury and emerging roles in the regulation of neuronal excitability, pharmacological targeting of these mechanisms may prove beneficial at reducing PTE. Further pre-clinical studies are needed to elucidate the exact mechanisms by which these and other inflammatory mediators initiate or perpetuate the process of epileptogenesis.

Several different animal models of PTE have been well studied over the past decade [235]. These experimental models have demonstrated that TBI results in both acute and chronic changes to the neuronal environment that likely contribute to epileptogenesis, such as hyperexcitable recurrent circuitry in the dentate gyrus of the hippocampus [235–237]. Whilst these models allow for the investigation of various mechanisms and testing of potential therapeutics, rodent models of PTE are not without limitations. Some models can produce a high level of epileptiform activity when measured with electroencephalogram, but most models are limited in the amount of spontaneous seizure activity they induce, leading to the need for higher animal numbers [11, 238]. One study that used a controlled cortical impact model of PTE, found that only 20–36% of animals given a severe TBI developed spontaneous behavioral seizures [239]. Though this presents logistical challenges, this incidence is only slightly higher than the estimated incidence in the clinical setting, which one study found to be 16.7% for severe TBI [240]. Animal models are also notoriously time-consuming as the latency to spontaneous seizure onset can be weeks to months after the initial injury [238, 241]. To increase the number of animals that can be investigated in relation to epileptogenesis and combat time restraints, some studies incorporate the administration of a pro-convulsant agent, such as pentylenetetrazol (PTZ), to unveil changes to seizure threshold as a surrogate marker of seizure susceptibility [235, 238, 241]. Many neuropathological processes that occur following TBI, including the neuroinflammatory response, can also differ greatly due to the developmental state of the brain at the time of impact [1]. This confound should be taken into account when investigating both the underlying mechanisms of epileptogenesis and piloting novel therapeutic interventions. Despite these limitations, these models show high clinical relevance and are useful tools with which to study PTE, and they are continually being improved upon.

Much investigation of neuroinflammatory intervention as a therapeutic option after TBI has focused on broad-target agents, such as minocycline, erythropoietin, and progesterone. All of these treatments were proposed to reduce neuroinflammation after TBI, generating promising results in pre-clinical research but failing to produce short-term improvement after TBI during clinical trials, as reviewed elsewhere [242]. However, these treatments, which are both pleiotropic in nature and relatively safe for long-term use, may have more success in relation to the chronic outcomes such as PTE as many of the effects of neuroinflammation persist long after the offending insult, yet few studies have considered the potential effects of anti-inflammatory treatments on long-term epileptogenesis after injury. Hypothermia is another broad-target treatment for neuroinflammation that has had some success in providing neuroprotection in the context of TBI and spinal cord injuries, with some evidence suggesting that it may also prevent epileptogenesis [243–245]. The potential to prevent or reduce PTE by targeting other inflammatory mediators in the post-injury neural environment, such as serum albumin and TGF-β, have not yet been explored. Additional potential targets include TNF-α, which has been shown to influence neurotransmission by altering excitatory post-synaptic currents and decreasing GABA-mediated inhibitory synaptic strength in hippocampal neurons [246]; the prostaglandin receptor EP2, which mediates COX-2 inflammatory signaling and appears to promote seizures by aggravating neuronal injury [247]; as well as factors involved in regeneration and regrowth such as fibroblast growth factor, tropomyosin receptor kinase B, and insulin-like growth factor-1. [248, 249]

## Conclusion

This review of the published literature has found that several inflammatory mediators, including IL-1β and HMGB1, exhibit epileptogenic and ictogenic properties, acting on glia and neurons both directly and indirectly to influence neuronal excitability. As neuroinflammation is a central component of the neuropathology after TBI, comparable mechanisms are likely to be involved in the process of epileptogenesis leading to PTE. An increased understanding of how inflammation influences epileptogenesis may reveal novel therapeutic targets and strategies to prevent or reduce seizures after TBI. As PTE is often resistant to existing pharmaceutical treatments [33, 34, 36], there is an urgent need for further research to develop preventative treatments to improve both the quality and longevity of life for TBI survivors.

## Abbreviations

ASDs: Anti-seizure drugs
AMPA: Alpha-amino-3-hydroxy-5-methyl-4-isoxazole-propoinate
BBB: Blood-brain barrier
CA3: Region III of hippocampus proper
CCL2: Chemokine (C-C Motif) ligand 2
CCL3: Chemokine (C-C Motif) ligand 3
CCL6: Chemokine (C-C Motif) ligand 6
CNS: Central nervous system
COX-2: Cyclooxygenase-2
CSF: Cerebrospinal fluid
CXCL8: Chemokine (C-X-C motif) ligand 8
DAMP: Damage associated molecular pattern
EC: Endothelial cells
EP2: Receptor for prostaglandin E2
FS: Febrile seizures
GABA: γ-aminobutyric acid
HMGB1: High mobility group box 1 protein
IL-1R: IL-1 receptor
IL-1R1: Cell surface type 1 IL-1 receptor
IL-1α: Interleukin-1α
IL-1β: Interleukin-1β
IL-6: Interleukin-6
IL-10: Interleukin-10
IL-12: Interleukin-12
IRF3: Interferon regulatory transcription factor 3
MAPK: Mitogen-activated protein kinase
MyD88: Myeloid differentiation primary response gene 88
NF-κB: Nuclear factor Kappa-light-chain-enhancer of activated B cells
NMDA: N-methyl-d-aspartate
p7: Postnatal day 7
p28: Postnatal day 28
PTE: Post-traumatic epilepsy
PTZ: Pentylenetetrazol
ROS: Reactive oxygen species
SNPs: Single nucleotide polymorphisms
TBI: Traumatic brain injury
TGF-β: Transforming growth factor-β
TLE: Temporal lobe epilepsy
TLR: Toll-like receptor
TNF-α: Tumor necrosis factor- α
TRAF6: TNF receptor associated factor 6
